# Taxonomic studies on the genus *Ectatosticta* (Araneae, Hypochilidae) from China, with descriptions of two new species

**DOI:** 10.3897/zookeys.954.52254

**Published:** 2020-07-29

**Authors:** Yejie Lin, Shuqiang Li

**Affiliations:** 1 Hebei Key Laboratory of Animal Diversity, College of Life Science, Langfang Normal University, Langfang 065000, China; 2 Institute of Zoology, Chinese Academy of Sciences, Beijing 100101, China; 3 Chinese Academy of Sciences

**Keywords:** diagnosis, etymology, taxonomy, type, webs

## Abstract

Species of the spider family Hypochilidae Marx, 1888 from China are studied, including two known species and two new species of the genus *Ectatosticta* Simon, 1892. The new species are *E. wukong***sp. nov.** (♂♀) from Sichuan and *E. xuanzang***sp. nov.** (♀) from Tibet.

## Introduction

Hypochilidae Marx, 1888 is a small family that includes two genera: *Hypochilus* Marx, 1888 and *Ectatosticta* Simon, 1892. *Hypochilus* is endemic to the USA and includes ten species, whereas *Ectatosticta* is endemic to China and until now only included two species: *E. davidi* (Simon, 1889) from Shaanxi and *E. deltshevi* Platnick & Jäger, 2009 from Qinghai (WSC 2020, Li 2020).

Hypochilidae was considered the sister group of all other araneomorph spiders (Platnick 1977), but Wheeler et al. (2017) confirmed that Hypochilidae is the sister group of Filistatidae Simon, 1864. Unlike *Hypochilus*, *Ectatosticta* build simple sheet webs between soil blocks, huge rocks or in tree trunks. On one side of the web of some species there is a tube-retreat which typically extends into rock crevices, soil or between roots.

In this paper, photographs of two known *Ectatosticta* species are provided, of which *E. davidi* (Simon, 1889) is based on material collected near the type locality and *E. deltshevi* Platnick & Jäger, 2009 is based on the male holotype and females from the same locality as the holotype. In addition, two new species of the genus *Ectatosticta* are described: *E. wukong* sp. nov. (♂♀) from Sichuan and *E. xuanzang* sp. nov. (♀) from Tibet.

## Material and methods

All specimens were preserved in 75% ethanol. Female genitalia were cleared in a trypsin enzyme solution to dissolve non-chitinous tissue. Specimens were examined under a LEICA M205C stereomicroscope. Photomicroscope images were taken with an Olympus C7070 zoom digital camera (7.1 megapixels). Photos were stacked with Helicon Focus 6.7.1 (Khmelik et al. 2006) and processed in Adobe Photoshop CC 2018.

All measurements are in millimeters. Eye sizes are measured as the maximum diameter from either the dorsal or frontal view. Leg measurements are given as follows: total length (femur, patella + tibia, metatarsus, tarsus). Distribution maps were generated using ArcMap software 10.2 (ESRI 2002).

Abbreviations:

**ALE** anterior lateral eyes

**AME** anterior median eyes

**C** conductor

**E** embolus

**IS** inner spermathecae

**OS** outer spermathecae

**PLE** posterior lateral eyes

**PME** posterior median eyes

**S** spermathecae

**TS** thickened setae

The material studied in the paper is housed in the Institute of Zoology, Chinese Academy of Sciences (**IZCAS**) in Beijing, China.

## Taxonomy

### Family Hypochilidae Marx, 1888

#### Genus Ectatosticta

Taxon classificationAnimaliaAraneaeHypochilidae

Simon, 1892

2805C737-4C67-5A2A-91B3-55FE832FDA0F

##### Type species.

*Hypochilus davidi* Simon, 1889 from China.

##### Diagnosis.

*Ectatosticta* can be easily distinguished from *Hypochilus* by the rectangular labium which is almost as long as wide and bears a pair of triangular posterolateral flanges, also by numerous leg spines (Forster et al. 1987) and in the lateral view of the male palp, the cymbium to bulb length ratio is almost 3:1 (Figs 2, 4) but nearly 1 : 1 in *Hypochilus* (Forster et al. 1987: figs 38, 43, 48, 53, 58, 63, 68, 73).

##### Distribution.

China.

### Key to *Ectatosticta* males

**Table:** 

1	Male palp with fewer than 5 thickened setae, the most dorsal setae are dispersed, and the length ratio of the embolus to the embolus base is more than 2 : 1 (Fig. 1)	**2**
–	Male palp with 5–7 thickened setae, all closely appressed one another, and the length ratio of the embolus to the embolus base is almost 1 : 1 (Fig. 1)	***E. davidi***
2	Male palp with 4 thickened setae, the dorsalmost setae are dispersed and the length ratio of the embolus to the embolus base is almost 2 : 1 (Fig. 1)	***E. deltshevi***
–	Male palp with 2 thickened setae, the length ratio of the embolus to the embolus base is almost 3 : 1 (Fig. 1)	***E. wukong* sp. nov.**

### Key to *Ectatosticta* females

**Table:** 

1	Two pairs of spermathecae (Fig. 5A, B, D)	**2**
–	One pair of spermathecae (Fig. 5C)	***E. wukong* sp. nov.**
2	The ratio of the length of the inner spermathecae to the outer spermathecae is almost 1 : 3 (Fig. 5D)	***E. xuanzang* sp. nov.**
–	The ratio of the length of the inner spermathecae to the outer spermathecae is almost 1 : 1 to 1 : 2 (Fig. 5A, B)	**3**
3	Spermathecae weakly sclerotized (Fig. 5A)	***E. davidi***
–	Spermathecae strongly sclerotized (Fig. 5B)	***E. deltshevi***

#### Ectatosticta davidi

Taxon classificationAnimaliaAraneaeHypochilidae

(Simon, 1889)

5B625907-1C1E-54E7-A908-93EB6C7F6C3F

**Figure 1.**
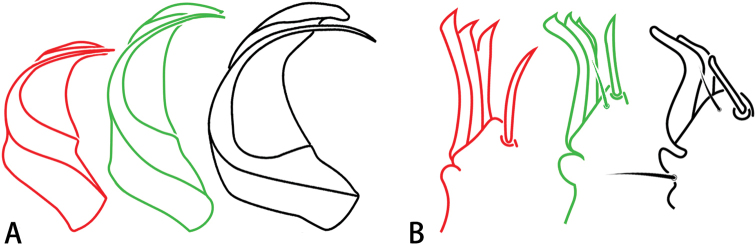
*Ectatosticta* spp., outlines of male bulbs and thickened setae in retrolateral view (Red line, *E. davidi*; green line, *E. deltshevi*, holotype; black line, *E. wukong* sp. nov., holotype) **A** bulbs **B** thickened setae.

**Figure 2.**
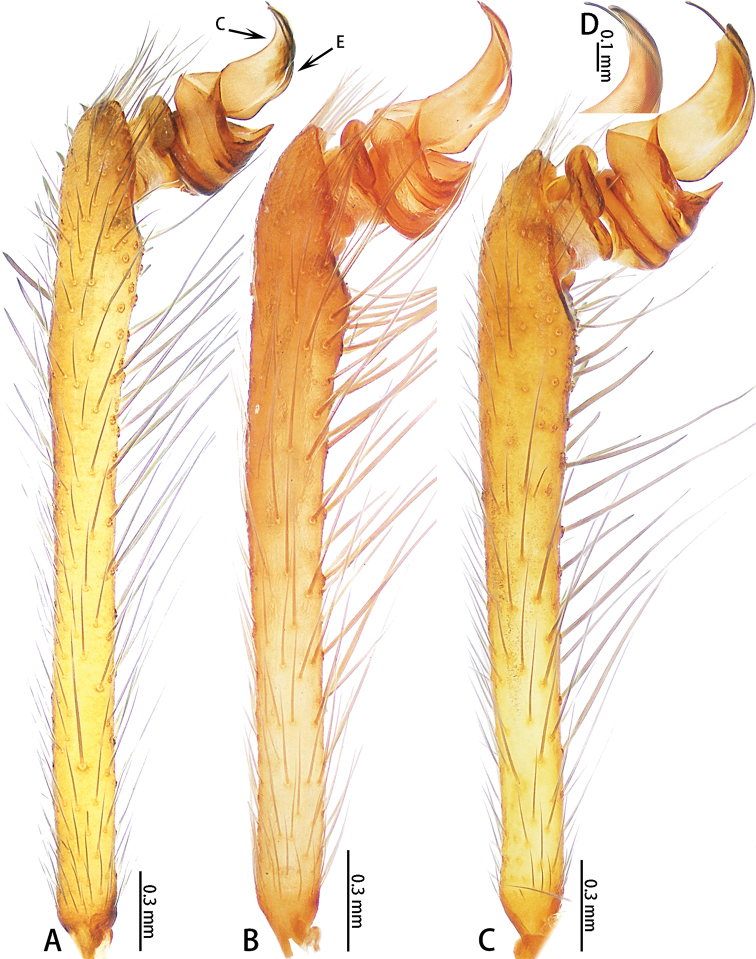
*Ectatosticta* spp., prolateral view of left male palps **A***E. davidi*, male from Shaanxi **B***E. deltshevi*, holotype **C***E. wukong* sp. nov., holotype **D***E. wukong* sp. nov., embolus and conductor of right palp (rotated horizontally), holotype.

**Figure 3.**
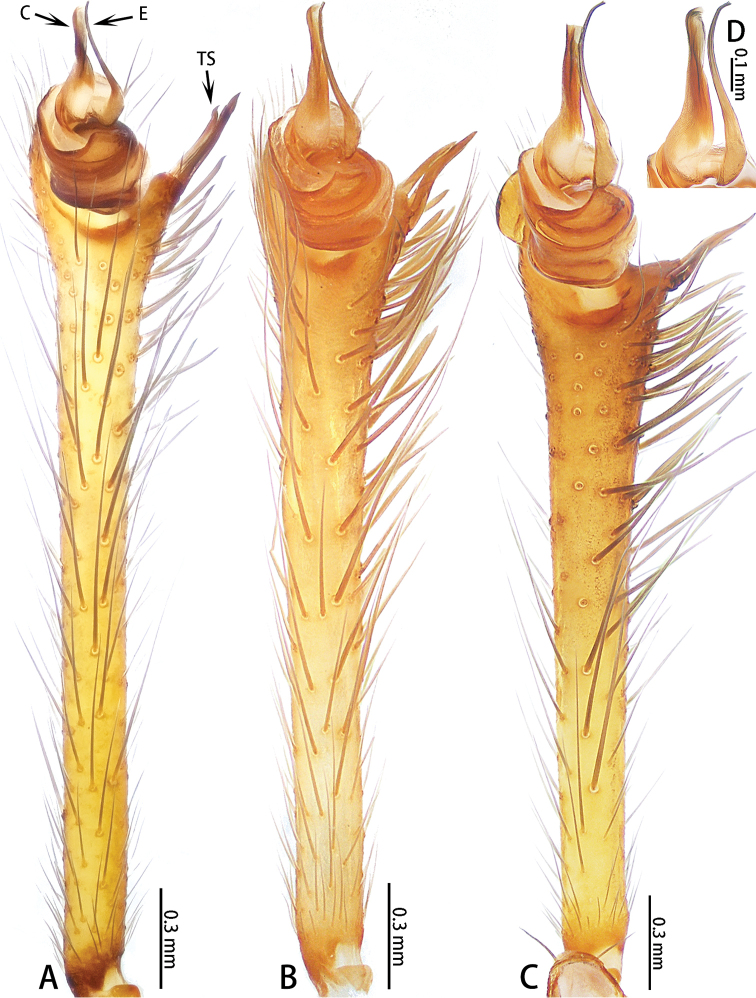
*Ectatosticta* spp., ventral view of left male palps **A***E. davidi*, male from Shaanxi **B***E. deltshevi*, holotype **C***E. wukong* sp. nov., holotype **D***E. wukong* sp. nov., embolus and conductor of right palp (rotated horizontally), holotype.

**Figure 4.**
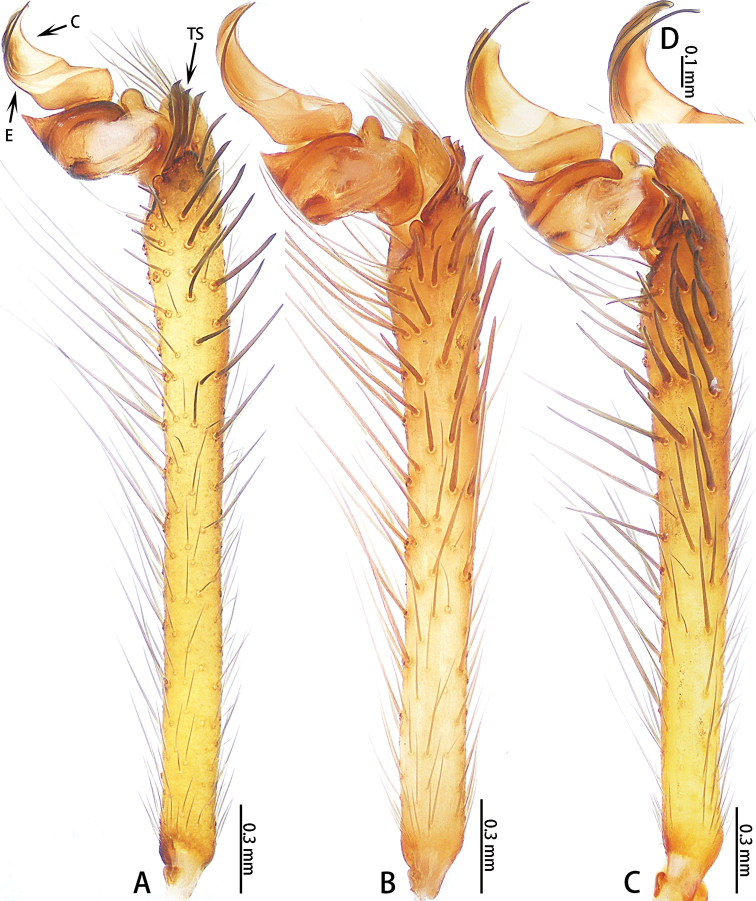
*Ectatosticta* spp., retrolateral view of left male palps **A***E. davidi*, male from Shaanxi **B***E. deltshevi*, holotype **C***E. wukong* sp. nov., holotype **D***E. wukong* sp. nov., embolus and conductor of right palp (rotated horizontally), holotype.

**Figure 5.**
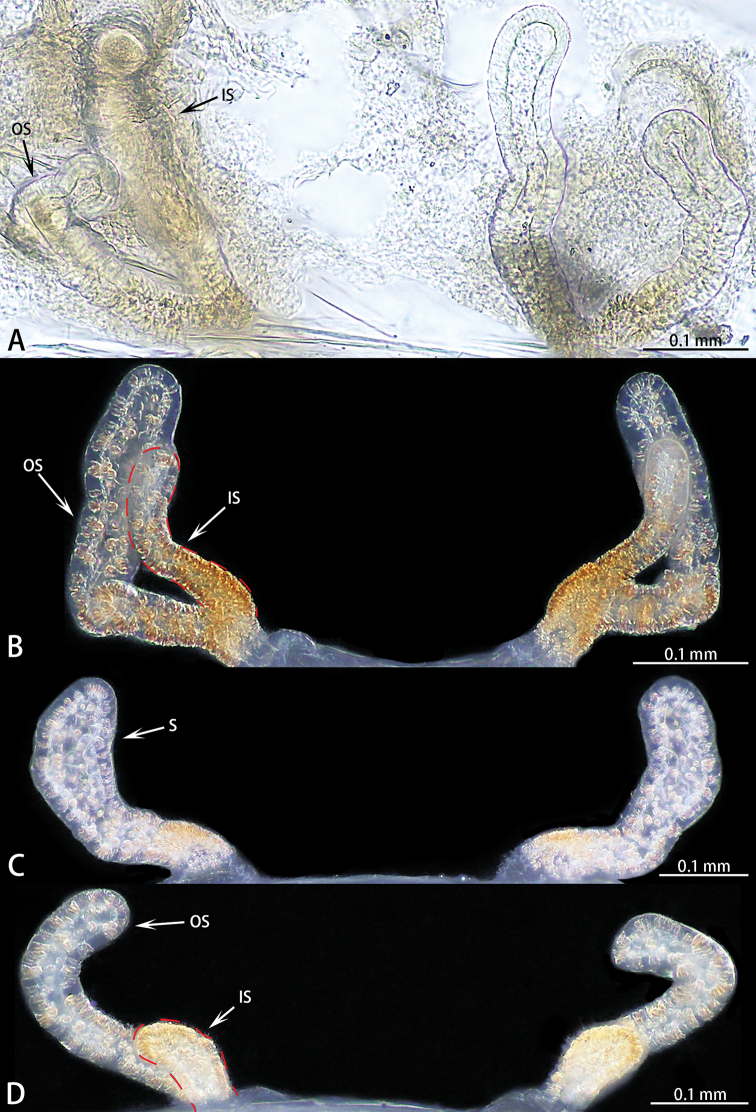
*Ectatosticta* spp., dorsal view of female genitalia **A***E. davidi*, female from Shaanxi **B***E. deltshevi*, female from Qinghai (type locality) **C***E. wukong* sp. nov., paratype **D***E. xuanzang* sp. nov., holotype.

**Figure 6.**
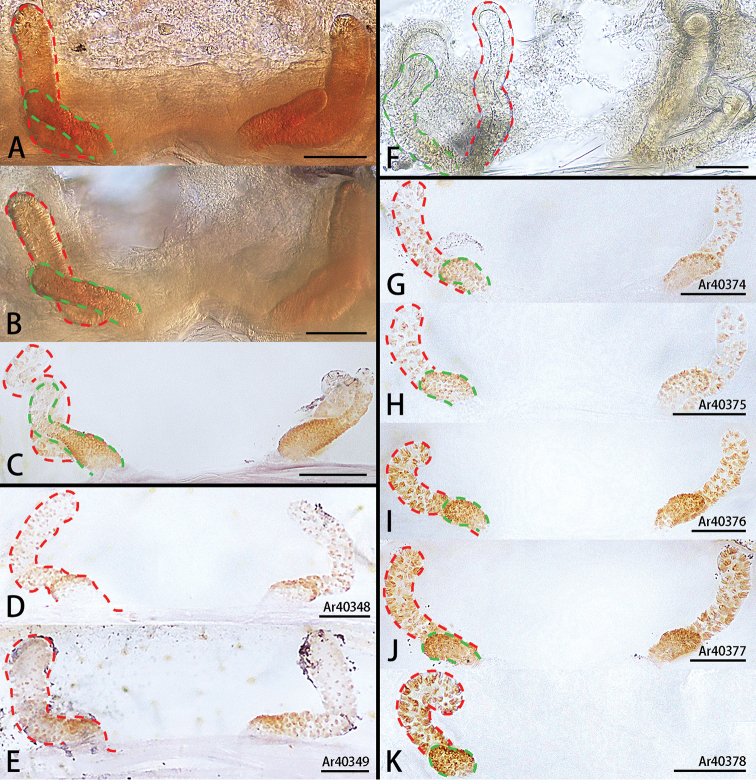
*Ectatosticta* spp., variation of female genitalia (red line, inner spermathecae (**A–C**, **F–K**) or spermathecae (**D, E**); green line, outer spermathecae) **A–C***E. deltshevi*, females from Qinghai (type locality) **D, E***E. wukong* sp. nov., paratypes **F***E. davidi*, females from Shaanxi **G–K***E. xuanzang* sp. nov., paratypes. Scale bars: 0.1 mm.

Figs 1 , 2A , 3A , 4A , 5A , 6F , 8

Hypochilus davidi Simon 1889: 208; Simon 1892: 204, figs 143–146, 148, 149; Gertsch 1958: 13, figs 10, 19, 22–31; Lehtinen, 1967: 431, fig. 15; Platnick and Jäger 2009: 210, figs 1–4; Zhang and Wang 2017: 311, fig. 4f.

##### Type material.

***Syntypes*** 1♂ 1♀, Muséum national d’Histoire naturelle, Paris, label reads “Inkiaphou, Chine méridionale”, which should be on Mt. Qinling in Shaanxi Province (see Platnick and Jäger 2009: Yinjiapo or Yinjiapu, now known as Yonxingcun in Xi'an City, Huyi District, Laoyu Town, 33.98232N, 108.52079E), not examined.

##### Other material examined.

1♂, China, Shaanxi Province, Chang’an, Xiaoyuhecun, Qiaotouchi, 02.V.2020, Jiazhou Lu leg.; 1♀ (IZCAS), China, Shaanxi Province, Mt. Taibaishan, above Houshenzi, tree line, scattered mixed coniferous/*Rhododendron* forest, 33.9122N, 107.7789E, 12–15.VI.1997, elevation ca. 3050 m, Peter Jäger leg.

##### Distribution.

China (Shaanxi).

#### Ectatosticta deltshevi

Taxon classificationAnimaliaAraneaeHypochilidae

Platnick & Jäger, 2009

B39B5DAD-9E59-5FD5-9759-C96A77328268

Figs 1 , 2B , 3B , 4B , 5B , 6A–C , 7A, B , 8

Ectatosticta davidi Li & Zhu, 1984: 510, figs A–G; Forster et al. 1987: 23, figs 6–16, 18–20, 23, 24, 31–36, 78–82; Song et al. 1999: 41, figs 11D, 17Q–T; Hu 2001: 69, figs 1.1–6; Song et al. 2001: 64, fig. 24A–E. All misidentified.
Ectatosticta deltshevi Platnick & Jäger, 2009: 214.

##### Type material.

***Holotype*** ♂ (IZCAS-Ar28579), China, Qinghai Province, Huangyuan County, 15.IX.1984, Zhongshan Li leg., examined.

##### Other material examined.

2♂2♀ (IZCAS), China, Qinghai Province, Huangyuan County, 15.IX.1984, Zhongshan Li leg.; 2♀ (IZCAS), China, Qinghai Province, Haidong, Huzhutu Autonomous County, Jinchuan County, Jiading, Beishan National Park, 36.9378N, 102.4575E, elevation ca. 2442 m, 30.X.2019, Yejie Lin leg.

##### Distribution.

China (Qinghai).

##### Natural history.

Living in simple sheet webs between soil blocks or tree roots. On one side of the web there is tube-retreat that extends into the soil.

#### Ectatosticta wukong sp. nov.

Taxon classificationAnimaliaAraneaeHypochilidae

BD06E8C9-62B5-5ECF-93FB-D7A98A748BDD

http://zoobank.org/4BDB5B2E-0307-4B5C-B678-2C45F70762AD

Figs 1 , 2C, D , 3C, D , 4C, D , 5C , 6D , 6E , 8

##### Type material.

***Holotype*** ♂ (IZCAS-Ar40346), China, Sichuan Province, Hongyuan County, Shuajingsi, Mt. Zhegu to Shuamalukou, 31.9272N, 102.6546E, elevation ca. 3458 m, 23.XI.2019, Zhigang Chen leg. ***Paratypes*** 3♀ (IZCAS-Ar40347–Ar40349), same data as holotype.

##### Etymology.

The species is named after Wukong, a character in the classic Chinese novel *Journey to the West*, noun. *Journey to the West* was written during the Ming Dynasty (1368–1644 A.D) and is about the adventures of a priest, Xuanzang, and his three disciples, Wukong, Wuneng, and Wujing, as they travel west in search of the Buddhist Sutra. Their travel begins at what is today Xi'an (near the type locality of *E. davidi*), via Qinghai (close to the type locality of *E. deltshevi*), to South Xinjiang, Tibet (near the type locality of *E. xuanzang* sp. nov.) and India.

##### Diagnosis.

Males of *E. wukong* sp. nov. can be distinguished by having only two thickened setae retrolaterally on the cymbium and the length ratio of the embolus to the embolus base is almost 3 : 1 (Fig. 3C, D). Females can be distinguished by having one pair of spermathecae (Figs 5C, 6D, E).

##### Description.

**Male**: Total length 9.29, carapace 5.58 long, 3.14 wide, opisthosoma 4.40 long, 3.14 wide. Eye sizes and interdistances: AME 0.19, ALE 0.26, PME 0.23, PLE 0.24, AME–AME 0.16, AME–ALE 0.21, PME–PME 0.36, PME–PLE 0.10, AME–PME 0.07, ALE–PLE 0.02. Clypeus height 0.30. Chelicerae with seven promarginal and six retromarginal teeth. Leg measurements: leg I: 40.37 (11.60 + 12.88 + 9.42 + 6.47), leg II: 31.79 (9.10 + 10.51 + 7.95 + 4.23), leg III: 24.98 (7.24 + 8.64 + 5.70 + 3.40), leg IV: 32.53 (9.55 + 10.13 + 8.40 + 4.45). Leg formula: 1423.

Male palp (Figs 2C, D, 3C, D, 4C, D) simple, cymbium long, retrolaterally with an apophysis divided into two parts: a small, semicircular lobe with a seta and a large lobe with two strong setae placed closely together. Embolus thin, length ratio of embolus to embolus base 3:1. Conductor sickle-shaped.

##### Female.

Total length 10.77, carapace 4.70 long, 3.28 wide, opisthosoma 6.79 long, 4.87 wide. Eye sizes and interdistances: AME 0.17, ALE 0.26, PME 0.23, PLE 0.29, AME–AME 0.18, AME–ALE 0.28, PME–PME 0.36, PME–PLE 0.27, AME–PME 0.06, ALE–PLE 0.07. Clypeus height 0.36. Chelicerae with seven promarginal and six retromarginal teeth. Leg measurements: Leg I: 29.10 (8.40 + 10.00 + 6.60 + 4.10), leg II: 25.44 (6.99 + 8.91 + 5.90 + 3.64), leg III: 18.73 (5.64 + 6.15 + 4.35 + 2.59), leg IV: 23.92 (7.31 + 7.50 + 5.83 + 3.28). Leg formula: 1243.

Female genitalia (Figs 5C, 6D, E) simple, one pair of spermathecae, spermathecae slightly curved.

##### Distribution.

Known only from the type locality.

#### Ectatosticta xuanzang sp. nov.

Taxon classificationAnimaliaAraneaeHypochilidae

AD8B7D44-D710-5F24-85FD-C90139F8C4D8

http://zoobank.org/3A050541-598F-4349-8B86-C21E11F5B0CB

**Figure 7.**
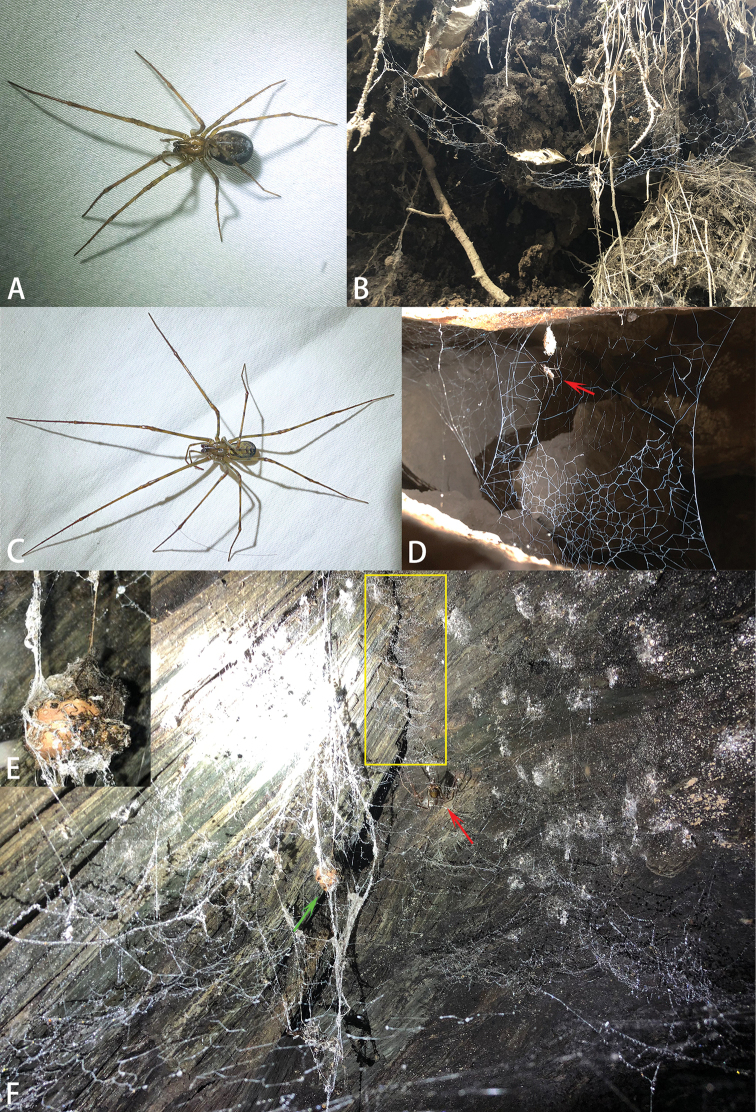
Photos of live *Ectatosticta* spp. **A***E. deltshevi*, female from Qinghai **B***E. deltshevi* and web **C***E. xuanzang* sp. nov., holotype from Tibet **D***E. xuanzang* and web **E** Egg sac **F** A typical web of *Ectatosticta*. Egg sac marked with green arrow, spider marked with red arrow and assembled nymphs marked with yellow rectangle.

**Figure 8.**
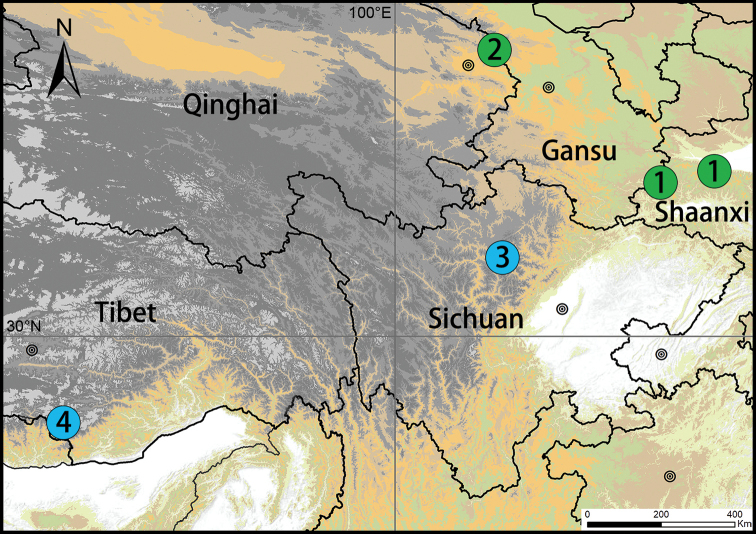
Distribution records of *Ectatosticta* species from China. **1***E. davidi***2***E. deltshevi***3***E. wukong* sp. nov. **4***E. xuanzang* sp. nov.

Figs 5D , 6G–K , 7C, D , 8

##### Type material.

***Holotype*** ♀(IZCNS-Ar40373), China, Tibet Autonomous Region, Lhoka, Cona County, Marmang, Lebugou, Yelang Valley, 27.8682N, 91.8110E, elevation ca. 3118 m, 12.X.2019, Yejie Lin leg. ***Paratypes*** 5♀ (IZCNS-Ar40374–Ar40378), same data as holotype.

##### Etymology.

The species is named after Xuanzang, a character in the classic Chinese novel *Journey to the West*, noun.

##### Diagnosis.

Females of *E. xuanzang* sp. nov. can be distinguished by the ratio of the length of the inner spermathecae to the outer spermathecae of almost 1:3 (Figs 5D, 6G–K) (vs. almost 1:1 in *E. davidi* and 1:2 to 1:1 in *E. deltshevi* (Figs 5A, B, 6A–C, F)) and the ratio of leg I length to the carapace length is almost 1:8 (vs. almost 1:6 in *E. wukong* sp. nov. and *E. deltshevi* and 1.7 in *E. davidi*).

##### Description.

**Female**. Total length 12.59, carapace 6.03 long, 3.60 wide, opisthosoma 6.22 long, 4.40 wide. Eye sizes and interdistances: AME 0.15, ALE 0.31, PME 0.29, PLE 0.28, AME–AME 0.19, AME–ALE 0.37, PME–PME 0.42, PME–PLE 0.27, AME–PME 0.07, ALE–PLE 0.06. Clypeus height 0.45. Chelicerae with seven (n = 3) or eight (n = 3) promarginal and 6–9 (6(n = 1), 7(n = 4), 9(n = 1)) retromarginal teeth. Leg measurements: Leg I: 51.47 (15.45 + 16.28 + 12.95 + 6.79), leg II: 47.88 (14.03 + 16.22 + 11.60 + 6.03), leg III: 37.66 (11.67 + 12.50 + 9.04 + 4.45), leg IV: 44.23 (12.31 + 14.49 + 12.11 + 5.32). Leg formula: 1243.

Female genitalia (Figs 5D, 6G–K) simple, with two pairs of slightly curved spermathecae. Inner spermathecae small, outer spermathecae curved, three times the length of inner spermathecae.

##### Male.

Unknown.

##### Distribution.

Known only from the type locality.

##### National history.

In damp rocky areas, hiding between huge stones. They build simple sheet webs without a tube-retreat.

## Discussion

Platnick & Jäger (2009) pointed out that the number of thickened setae in males of *Ectatosticta deltshevi* was four, whereas in *E. davidi* it was five to seven. However, it is necessary to examine more male specimens to learn more about the extent of variation. Based on the examination of all female specimens available, the extent of sclerotization of the spermathecae seems stable within the species. This study is currently being expanded to include molecular data and additional specimens from southwestern China and the Himalayas which will continue to increase our knowledge of *Ectatosticta*.

## Supplementary Material

XML Treatment for Ectatosticta

XML Treatment for Ectatosticta davidi

XML Treatment for Ectatosticta deltshevi

XML Treatment for Ectatosticta wukong

XML Treatment for Ectatosticta xuanzang
